# Disentangling Detoxification: Gene Expression Analysis of Feeding Mountain Pine Beetle Illuminates Molecular-Level Host Chemical Defense Detoxification Mechanisms

**DOI:** 10.1371/journal.pone.0077777

**Published:** 2013-11-01

**Authors:** Jeanne A. Robert, Caitlin Pitt, Tiffany R. Bonnett, Macaire M. S. Yuen, Christopher I. Keeling, Jörg Bohlmann, Dezene P. W. Huber

**Affiliations:** 1 Ecosystem Science and Management Program, University of Northern British Columbia, Prince George, BC, Canada; 2 Michael Smith Laboratories, University of British Columbia, Vancouver, BC, Canada; Natural Resources Canada, Canada

## Abstract

The mountain pine beetle, *Dendroctonus ponderosae*, is a native species of bark beetle (Coleoptera: Curculionidae) that caused unprecedented damage to the pine forests of British Columbia and other parts of western North America and is currently expanding its range into the boreal forests of central and eastern Canada and the USA. We conducted a large-scale gene expression analysis (RNA-seq) of mountain pine beetle male and female adults either starved or fed in male-female pairs for 24 hours on lodgepole pine host tree tissues. Our aim was to uncover transcripts involved in coniferophagous mountain pine beetle detoxification systems during early host colonization. Transcripts of members from several gene families significantly increased in insects fed on host tissue including: cytochromes P450, glucosyl transferases and glutathione S-transferases, esterases, and one ABC transporter. Other significantly increasing transcripts with potential roles in detoxification of host defenses included alcohol dehydrogenases and a group of unexpected transcripts whose products may play an, as yet, undiscovered role in host colonization by mountain pine beetle.

## Introduction

The mountain pine beetle, *Dendroctonus ponderosae* (Coleoptera: Curculionidae), is a native species of bark beetle that caused unprecedented damage to the pine forests of British Columbia and other parts of western North America, and is currently expanding its range into the boreal forests of central and eastern Canada [1]–[2]. Large areas of susceptible host trees and warmer winters [3] have caused this insect and its fungal associates to affect an estimated 18.1 million hectares of forest (predominantly lodgepole pine, *Pinus contorta*) in British Columbia [4]. The devastation of large areas of pine in British Columbia has impacted the sustainability of the timber harvesting industry [5]–[6] and may also potentially affect the viability of relatively new industries such as wood pellet production for bioenergy. In addition, mountain pine beetles have recently moved from British Columbia’s lodgepole pine forests into a new host, jack pine, *Pinus bankisana*, in the forests of Alberta [1]. It is still unclear how this insect will fare in the new host species, specifically whether detoxification mechanisms adapted to its current hosts will be as effective as it moves to this newer host. Research into the current mountain pine beetle epidemic can provide crucial information for predicting and managing timber and bioenergy feedstock supply into the future, as well as illuminating the potential for this insect to move through the boreal forest, comprised largely of jack pine, across Canada.

Adult mountain pine beetle host selection and subsequent successful colonization of host tissue is fraught with challenges including copious toxic host defense mechanisms. Host conifers are saturated with potentially toxic specialized metabolites that insects must tolerate or detoxify in order to successfully reproduce [7]–[8]. During beetle population outbreaks, host defenses are overwhelmed by mass attacks where, using powerful pheromone aggregation signals, large numbers of insects are induced to simultaneously attack a host tree. Beetle-vectored pathogenic fungi inoculated during attack may also aid in overcoming host defences [9]–[10], and contribute to the death of the host tree (for example, see [11]). After successful mass attack, adults excavate egg galleries in the resin-saturated phloem and the larvae must feed and develop on toxic tissues in order to survive the winter [12]. Resistance or tolerance to host specialized metabolites are key factors in mountain pine beetle reproductive success.

We used RNA-seq analysis to monitor gene expression patterns of mountain pine beetle adult males and females during early colonization of lodgepole pine in order to investigate potential molecular-level host chemical detoxification mechanisms. Metabolic changes occurring shortly after host colonization suggest a stress response in adult mountain pine beetles, including physiological priming for detoxification as well as preparation for reproduction. We uncovered transcript changes in several groups of enzymes that are likely to be important in the host chemical detoxification mechanisms of mountain pine beetle. These included cytochromes P450, glucosyl transferases and glutathione S-transferases, esterases, alcohol dehydrogenases, and ABC transporters; as well as several gene transcripts implicated in immune system responses, reproduction, pheromone flux, and digestion.

## Materials and Methods

We conducted an RNA-seq analysis of mountain pine beetle adults fed on susceptible host material versus starved adults over a 24-hour period. Differential gene expression from large-scale transcriptomics analysis using an Illumina-based platform was used to identify gene candidates that may be involved in host colonization physiology, including specialized metabolite detoxification.

### Insect Origins

Lodgepole pine bolts infested with *D. ponderosae* were harvested from an area near to Penticton, British Columbia, with outbreak population levels of insects in May of 2010 (UTM: 5478504 northing, 321764 easting, zone 11 U). Bolts were provided by Doug Batemen Logging from the company harvest for that area; no further permissions were required. The collection of bolts did not involve endangered or protected species, and ethics approval is not required by an Animal Care and Use Committee for research on insects. Bolt ends were waxed with paraffin to prevent drying and to ensure optimal beetle development. During late-instar larval development, bolts were contained in vented plastic storage bins at ambient outdoor spring and summer temperatures and were misted with water every few days. During beetle emergence (July through August 2010), cages were checked daily to collect emerged mountain pine beetle adults. The insects were separated by sex according to [13] and were stored at 4°C in petri dishes containing lightly moistened Kimwipes. Beetles were stored for a maximum of 10 days prior to use in experiments and were checked to ensure a lack of storage-related damage prior to use.

### Feeding Treatments

As we were interested in changes in transcript levels for adult female and male insects feeding on host plant tissue, we used two treatments: insects starved for 24 hours (control), and insects fed in male-female pairs on host tissues for 24 hours (treatment).

The control beetles were kept in the dark, at room temperature, for 24 hours and those insects were maintained individually in 1.5 mL microcentrifuge tubes with a small hole in the lid. After 24 hours, the insects were flash frozen in liquid nitrogen and stored at −80°C until RNA extraction.

Simultaneously, the treatment insects were placed into the phloem tissue of freshly cut (less than 24 hours before the experiment began) and waxed bolts of lodgepole pine. After drilling a small entrance hole (approximately 3 mm in diameter), insects were placed under the bark in randomly chosen pairs of females and males. Females were placed under the bark first, followed by the males. Insects were held in the holes by wire mesh stapled to the outside of the bark. The treatment insects were allowed to feed under the bark for 24 hours. We removed adults from galleries showing excavation of frass (an indication of feeding) and once again separated the insects into males and females. Beetles were then flash frozen in liquid nitrogen and stored at −80°C for subsequent RNA extraction.

### RNA Extraction

RNA extraction was performed with individual whole beetles using the MagMAX™-96 Total RNA Isolation Kit (Ambion). For each beetle, RNA quality was determined using an Experion Automated Electrophoresis Station (BioRad) without heating the extracted RNA to 70°C degrees because of the tendency for the ribosomal RNA 28S subunit band to break in some insects [14], including mountain pine beetle [15]. RNA quantity was determined using a Qubit 2.0 fluorometer (Invitrogen).

A minimum number of four high quality extractions [RNA integrity numbers (RIN) >7] were pooled in order to achieve the 10 µg total RNA required for library construction and RNA-seq analysis at Canada’s Michael Smith Genome Sciences Centre.

#### RNA-seq method

Samples were shipped on dry ice to Canada’s Michael Smith Genome Sciences Centre in Vancouver, BC for paired-end sequencing using the Illumina HiSeq 2000 system platform. Sixteen libraries were generated and indexed: four replicates each of starved females, fed females, starved males, and fed males. 50 bp sequences were requested, although 75 bp sequences were generated for some of the sequencing lanes because of advancing sequencing technologies. The 16 samples were multiplexed into four sequencing lanes, with one replicate of each treatment randomly assigned per lane so that every lane contained all four treatments.

### Data Analysis

Sequence read information was mapped to the gene models of the male mountain pine beetle genome sequence [16]. Sequence information was converted to fastq format using bam2fastq software (http://www.hudsonalpha.org/gsl/information/software/bam2fastq). The paired-end fastq files were mapped to the genome via CLC Genomics Workbench (http://www.clcbio.com/; CLC bio) using the parameters listed in Table S1. The raw RNA-seq data is available at the National Center for Biotechnology Information Sequence Read Archive (NCBI SRA) database (accession numbers SRS421461-64) under the TRIA umbrella BioProject (PRJNA169907) that identifies aggregated research project data generated from the TRIA research collaborations on mountain pine beetle systems genomics.

### Statistics

The RNA-Seq data were analysed using DESeq [17] package available from The R Project for Statistical Computing (www.r-project.org). The DESeq package calculates differential expression using a negative binomial distribution and a shrinkage estimator for the distribution’s variance. The program calculates fold changes for each gene as well as p-values adjusted (padj) for multiple comparisons using a Benjamini-Hochberg correction [18]. We used a 1% false discovery rate (padj <0.01) to identify transcript accumulation that was significantly different between treatments. The DESeq output is publicly available via figshare.

## Results

As we were interested in gene transcription changes of transcriptome profiles in MPB exposed to host tissue, we used two treatments in both sexes: starved insects versus 24 hours feeding on freshly cut lodgepole pine. RNA samples were analysed via paired-end sequencing using the Illumina HiSeq 2000 platform. Sixteen libraries were generated: four replicates each of starved females (libraries DEH01 to DEH04), fed females DEH05 to DEH08), starved males (DEH09 to DEH12), and fed males (DEH13 to DEH16). The modal sequence length in the cDNA libraries was approximately 250 base pairs. Only the pairs that mapped uniquely to one gene model on the genome were used in the analysis. This resulted in a uniform dataset containing approximately three to four million uniquely mapped pairs per library (Table 1). To identify differential transcript accumulation between treatments, we used p-values adjusted for multiple comparisons using a Benjamini-Hochberg correction with a 1% false discovery rate.

**Table 1:** Summary information for sequence data mapped to the male mountain pine beetle genome (13).

Treatment	Replicate	Library name	Read length	Total pairs mapped	Uniquely mapped pairs
Starved females	1	DEH01	50	5,122,574	3,958,698
	2	DEH02	75	11,028,522	4,358,131
	3	DEH03	75	9,733,440	3,723,858
	4	DEH04	75	10,965,492	4,313,831
Fed females	1	DEH05	50	12,514,004	4,938,352
	2	DEH06	75	10,211,068	4,053,784
	3	DEH07	75	8,530,756	3,409,236
	4	DEH08	75	11,079,602	4,366,865
Starved males	1	DEH09	50	8,587,908	3,352,665
	2	DEH10	75	6,977,712	2,741,611
	3	DEH11	75	8,941,136	3,592,428
	4	DEH12	75	9,143,262	3,716,843
Fed males	1	DEH13	50	10,322,094	4,046,344
	2	DEH14	75	8,829,776	3,465,502
	3	DEH15	75	9,315,596	3,640,538
	4	DEH16	75	10,775,090	4,184,473

**Figure 1:**
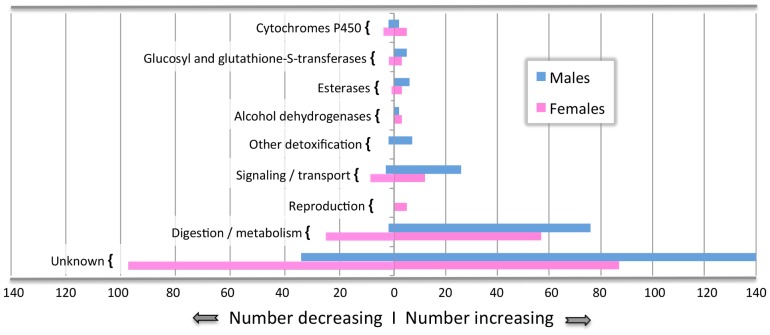
Annotated gene transcripts by functional category. The number of annotated genes within each putative functional category for male beetles (blue bars) and female beetles (pink bars) that had transcript levels either significantly increase or decrease (padj<0.01).

General functional categories for the transcripts showing significantly differential expression between the treatment and controls are shown in Figure 1. Increased transcript abundance was generally evident for functional categories involved in detoxification and reproduction. More specifically, we identified significantly changing transcript levels from gene families including cytochromes P450 (Table 2, Table 3), glutathione S-transferases (Table 4), esterases (Table 5), and one ABC transporter (Table 6). Other significantly increasing transcripts with potential roles in detoxification of host defenses include alcohol dehydrogenases (Table 7), oxidative stress and damage control transcripts (Table 8) as well as immune response gene expression (Table 9). In addition, other physiological processes that showed large changes in transcript accumulations included those involved in reproduction (Table 10), pheromone flux (Table 11), and digestive processes (Table 12). In each table, data generated from this experiment is compared to data from mountain pine beetle expressed sequence tag databases (EST databases) published in Keeling et al. 2012.

**Table 2:** Summary information for significantly (padj<0.01) up-regulated cytochromes P450 in fed versus starved females and males including the number of reads in each EST library 01 to14 with greater than 99% nucleotide identity.

MPB Genome gene model ID	Genbank accession number	Annotation	Fem	Male	Closest named sequence	GenBank accession number	01	02	03	04	05	06	07	08	09	10	11	12	13	14
			Fold chge	Fold chge								JH		MT	JH	JH	Fed	Fed	JH +MT	cold
							Larvae	Pupae	Pupae	Adult ant.	Adult	Adult	Adult	Adult	Adult Mid	Adult Mid	Adult Mid	Adult Mid	Adult head	Larvae
maker-Seq_1101813-augustus-gene-0.16	YQE_01868	CYP P450 6BQ5 [Tribolium castaneum]	–	136.9	CYP6DE4	JQ855671	–	–	–	–	–	–	–	–	2	4	1	–	2	2
maker-Seq_1101809-snap-gene-3.19	YQE_01841	CYP P450 CYP6BK17 [Tribolium castaneum]	65.6	–	CYP6DH1	JQ855674	–	–	–	–	–	–	–	1	–	–	2	–	–	1
augustus_masked-Seq_1096637-abinit-gene-0.0	YQE_00207	CYP P450 CY6BQ5 [Tribolium castaneum]	28.4	6.5	CYP6DK1	JQ855679	4	–	8	28	11	–	7	11	4	11	27	2	14	6
augustus_masked-Seq_1102694-abinit-gene-36.4	YQE_06277	CYP P450 [Tribolium castaneum]	7.7	–	CYP345F1	JQ855646	–	–	–	–	–	–	–	–	–	–	1	–	–	–
snap_masked-Seq_1102588-abinit-gene-0.30	YQE_04799	CYP P450 CY6BR3 [Tribolium castaneum]	7.5	–	CYP6DJ1	JQ855677	–	–	–	65	–	–	–	–	2	8	10	–	–	2
snap_masked-Seq_1102588-abinit-gene-0.33	YQE_04800	CYP P450 CYP6BR3 [Tribolium castaneum]	4.7	–	CYP6DJ2	JQ855678	–	–	–	–	–	–	–	–	1	10	4	–	13	–

* Abbreviations are as follows, Fem: females, ant: antennae only library, Adult mid: adult midgut and fatbody tissue only library, JH: juvenile hormone treated, MT: monoterpene treated, Fed: allowed to feed on lodgepole pine, cold: sampled during overwintering. For complete information on the contents of each EST library, please see Keeling et al. 2012.

**Table 3:** Summary information for significantly (padj<0.01) down-regulated cytochromes P450 in fed versus starved females and males including the number of reads in each EST library 01 to14 with greater than 99% nucleotide identity.

MPB Genome gene model ID	Genbank accession number	Annotation	Fem	Male	Closest named sequence	GenBank accession number	01	02	03	04	05	06	07	08	09	10	11	12	13	14
			Fold chge	Fold chge								JH		MT	JH	JH	Fed	Fed	JH +MT	cold
							Larvae	Pupae	Pupae	Adult ant.	Adult	Adult	Adult	Adult	Adult Mid	Adult Mid	Adult Mid	Adult Mid	Adult head	Larvae
snap_masked-Seq_1102963-abinit-gene-0.6	YQE_10907	CYP P450 CYP6BK17 [Tribolium castaneum]	−10.5	–	CYP6CR2	JQ855667	–	–	–	–	–	–	–	5	–	–	–	–	2	–
maker-Seq_1101741-snap-gene-13.46	YQE_01611	antennae-rich cytochrome P450 [Tribolium castaneum]	−5.7	–	CYP4BQ1	JQ855656	–	–	–	–	–	–	–	–	–	–	–	–	2	–
maker-Seq_1102716-augustus-gene-7.48	YQE_06842	cytochrome P450 9Z4 [Tribolium castaneum]	−5.2	–	CYP9AP1	JQ855681	–	–	–	2	–	–	–	1	3	2	3	–	3	–
maker-Seq_1103030-snap-gene-0.28	YQE_12529	antennae-rich cytochrome P450 [Tribolium castaneum]	−4.5	−2.7	CYP345E2	JQ855644	–	–	–	10	–	–	–	–	–	–	1	–	1	–
augustus_masked-Seq_1099578-abinit-gene-0.0	YQE_00788	antennae-rich cytochrome P450 [Tribolium castaneum]	–	−2.4	CYP6BW2	JQ855662	–	–	–	2	1	–	2	1	–	3	4	–	–	2

**Table 4:** Summary table for significantly (padj<0.01) increasing and decreasing glucosyl transferases in fed versus starved males and females including the number of reads in each EST library 01 to14 with greater than 99% nucleotide identity.

MPB Genome gene model ID	Accession number	Annotation	Females	Males	01	02	03	04	05	06	07	08	09	10	11	12	13	14
			Fold chge	Fold chge						JH		MT	JH	JH	Fed	Fed	JH +MT	cold
					Larvae	Pupae	Pupae	Adult ant.	Adult	Adult	Adult	Adult	Adult Mid	Adult Mid	Adult Mid	Adult Mid	Adult head	Larvae
maker-Seq_1103026-snap-gene-25.66	YQE_12426	Glucosyl glucuronosyl transferases [Tribolium castaneum]	–	4.7	–	–	–	–	–	–	–	–	–	–	–	–	–	–
genemark-Seq_1103037-abinit-gene-13.24	YQE_12751	Glucosyl glucuronosyl transferases [Tribolium castaneum]	2.6	3.8	–	–	–	–	–	–	–	–	–	2	3	–	–	–
maker-Seq_1102674-snap-gene-16.40	YQE_05665	Glucosyl glucuronosyl transferases [Tribolium castaneum]	2.6	5.0	–	–	–	–	–	–	–	–	2	–	–	–	–	2
snap_masked-Seq_1102275-abinit-gene-0.28	YQE_03117	glutathione S-transferase, [Pediculus humanus corporis]	2.3	2.4	–	–	–	–	7	2	–	1	7	–	13	1	–	2
snap_masked-Seq_1103037-abinit-gene-13.37	YQE_12753	Glucosyl glucuronosyl transferases [Tribolium castaneum]	–	2.3	–	–	–	–	1	–	–	–	–	2	4	1	–	4
genemark-Seq_1102383-abinit-gene-1.10	YQE_03750	antennal-enriched UDP-glycosyltransferase [Tribolium castaneum]	−4.5	–	–	–	–	–	–	–	–	2	–	–	–	–	–	–
genemark-Seq_1102383-abinit-gene-1.11	YQE_03749	Glucosyl glucuronosyl transferase [Culex quinquefasciatus]	−3.5	–	–	–	–	–	4	–	–	2	4	2	3	–	–	6

**Table 5:** Summary table for significantly (padj<0.01) increasing and decreasing esterases in fed versus starved males and females including the number of reads in each EST library 01 to14 with greater than 99% nucleotide identity.

MPB Genome gene model ID	Accession number	Annotation	Females	Males	01	02	03	04	05	06	07	08	09	10	11	12	13	14
			Fold chge	Fold chge						JH		MT	JH	JH	Fed	Fed	JH +MT	cold
					Larvae	Pupae	Pupae	Adult ant.	Adult	Adult	Adult	Adult	Adult Mid	Adult Mid	Adult Mid	Adult Mid	Adult head	Larvae
genemark-Seq_1102891-abinit-gene-0.13	YQE_09690	putative esterase [Tribolium castaneum]	9.3	–	–	–	–	–	–	4	–	2	10	13	13	4	–	–
genemark-Seq_1102308-abinit-gene-5.8	YQE_03254	lipase [Tribolium castaneum]	–	7.9	6	–	–	–	–	4	4	1	9	4	8	32	–	4
maker-Seq_1102308-snap-gene-5.25	YQE_03256	lipase [Tribolium castaneum]	–	5.8	–	–	–	–	–	–	–	–	–	16	32	4	-	8
maker-Seq_1102774-snap-gene-21.56	YQE_08627	alpha-esterase [Tribolium castaneum]	3.1	4.5	–	–	–	–	–	–	2	1	4	1	9	–	–	2
maker-Seq_1102417-snap-gene-2.48	YQE_03928	putative esterase [Tribolium castaneum]	–	2.6	–	1	–	–	–	–	–	1	–	–	–	–	–	0
maker-Seq_1102473-snap-gene-0.55	YQE_04217	carboxylesterase [Tribolium castaneum]	–	2.1	–	–	–	–	2	2	2	–	4	3	4	8	–	2
maker-Seq_1102432-augustus-gene-0.57	YQE_03970	lipase [Tribolium castaneum]	–	2.0	–	–	–	–	–	17	8	24	–	–	–	–	–	0
maker-Seq_1102308-snap-gene-6.52	YQE_03257	lipase [Tribolium castaneum]	−4.4	–	–	–	–	–	–	2	4	–	–	–	2	–	1	1
maker-Seq_1103039-snap-gene-0.57	YQE_12992	putative esterase [Tribolium castaneum]	−2.7	–	–	–	–	2	–	–	–	–	–	–	–	1	–	0

**Table 6:** Summary table for significantly (padj<0.01) increasing and decreasing ABC transporters in fed versus starved males and females including the number of reads in each EST library 01 to14 with greater than 99% nucleotide identity.

MPB Genome gene model ID	Accession number	Annotation	Females	Males	01	02	03	04	05	06	07	08	09	10	11	12	13	14
			Foldchge	Fold chge						JH		MT	JH	JH	Fed	Fed	JH +MT	cold
					Larvae	Pupae	Pupae	Adultant.	Adult	Adult	Adult	Adult	Adult Mid	AdultMid	Adult Mid	Adult Mid	Adult head	Larvae
maker-Seq_1102955-augustus-gene-1.33	YQE_10530	ABC transporter [Tribolium castaneum]	2.9	–	–	–	–	–	–	–	–	–	–	–	3	–	–	–

**Table 7:** Summary table for significantly (padj<0.01) increasing and decreasing alcohol dehydrogenases in fed versus starved males and females including the number of reads in each EST library 01 to14 with greater than 99% nucleotide identity.

MPB Genome gene model ID	Accession number	Annotation	Females	Males	01	02	03	04	05	06	07	08	09	10	11	12	13	14
			Fold chge	Fold chge						JH		MT	JH	JH	Fed	Fed	JH +MT	cold
					Larvae	Pupae	Pupae	Adult ant.	Adult	Adult	Adult	Adult	Adult Mid	Adult Mid	Adult Mid	Adult Mid	Adult head	Larvae
maker-Seq_1093767-snap-gene-0.4	YQE_00058	alcohol dehydrogenase [Tribolium castaneum]	6.5	5.0	–	–	–	–	–	–	–	–	–	1	1	–	–	–
maker-Seq_1101972-snap-gene-1.40	YQE_02573	alcohol dehydrogenase [Tribolium castaneum]	4.6	–	–	–	2	2	2	–	–	3	2	6	12	–	16	2
maker-Seq_1102727-augustus-gene-0.53	YQE_07092	alcohol dehydrogenase [Tribolium castaneum]	3.5	–	–	–	–	–	–	1	–	–	–	–	13	–	1	–
augustus_masked-Seq_1102997-abinit-gene-2.0	YQE_11466	zinc-containing alcohol dehydrogenase [Tribolium castaneum]	–	2.6	2	–	2	–	–	–	2	–	3	3	13	–	–	4

**Table 8:** Summary table for significantly (padj<0.01) increasing and decreasing transcripts implicated in damage control in fed versus starved males and females including the number of reads in each EST library 01 to14 with greater than 99% nucleotide identity.

MPB Genome gene model ID	Accession number	Annotation	Females	Males	01	02	03	04	05	06	07	08	09	10	11	12	13	14
			Fold chge	Fold chge						JH		MT	JH	JH	Fed	Fed	JH +MT	cold
					Larvae	Pupae	Pupae	Adult ant.	Adult	Adult	Adult	Adult	Adult Mid	Adult Mid	Adult Mid	Adult Mid	Adult head	Larvae
maker-Seq_1102995-snap-gene-0.45	YQE_11411	superoxide dismutase [Anopheles gambiae]	−5.3	−2.0	22	–	–	–	–	–	2	2	12	19	19	–	–	10
maker-Seq_1102594-augustus-gene-2.43	YQE_04823	DNA-damage inducible protein [Tribolium castaneum]	2.2	–	2	–	3	2	–	–	–	4	–	2	4	–	2	–
maker-Seq_1103012-augustus-gene-1.75	YQE_11784	proteasome subunit beta type 5,8 [Tribolium castaneum]	–	1.9	–	–	3	1	2	2	–	10	–	14	13	4	7	7
maker-Seq_1102687-augustus-gene-14.57	YQE_05916	thioredoxin-like protein [Tribolium castaneum]	–	1.8	2	–	18	33	8	2	4	12	8	10	15	–	12	8
genemark-Seq_1101822-abinit-gene-2.26	YQE_01952	luciferin-regenerating enzyme [Tribolium castaneum]	3.8	–	–	–	–	–	1	–	–	–	–	–	–	–	2	–

**Table 9:** Summary table for significantly (padj<0.01) increasing and decreasing anti-viral and anti-microbial immune response transcripts in fed versus starved males and females including the number of reads in each EST library 01 to14 with greater than 99% nucleotide identity.

MPB Genome gene model ID	Accession number	Annotation	Females	Males	01	02	03	04	05	06	07	08	09	10	11	12	13	14
			Fold chge	Fold chge						JH		MT	JH	JH	Fed	Fed	JH +MT	cold
					Larvae	Pupae	Pupae	Adult ant.	Adult	Adult	Adult	Adult	Adult Mid	Adult Mid	Adult Mid	Adult Mid	Adult head	Larvae
maker-Seq_1102721-augustus-gene-8.32	YQE_06950	salivary C-type lectin [Culex quinquefasciatus]	16.2	–	–	–	2	–	–	–	–	–	–	2	–	–	1	–
maker-Seq_1103023-augustus-gene-37.44	YQE_12213	lysostaphin [Staphylococcus simulans bv. Staphylolyticus]	–	3.2	2	–	–	–	2	–	2	–	–	–	–	–	–	2
maker-Seq_1102748-augustus-gene-3.55	YQE_07692	scavenger receptor class B, member 1-like [Tribolium castaneum]	2.3	2.7	–	–	–	6	–	–	1	–	–	–	–	–	–	–
maker-Seq_1103007-augustus-gene-11.40	YQE_07692	scavenger receptor [Tribolium castaneum]	–	2.2	–	–	–	–	–	–	–	–	–	1	2	–	–	–
maker-Seq_1103015-augustus-gene-1.33	YQE_11841	ebna2 binding protein P100 [Tribolium castaneum]	3.1	–	–	–	–	–	–	2	2	–	–	4	–	–	4	–
genemark-Seq_1102685-abinit-gene-4.14	YQE_05787	IFN-inducible and antiviral protein [Tribolium castaneum]	2.2	–	4	–	–	2	–	–	–	–	7	4	8	4	3	–

**Table 10:** Summary table for significantly (padj<0.01) increasing and decreasing transcripts implicated in reproductive physiology in fed versus starved males and females including the number of reads in each EST library 01 to14 with greater than 99% nucleotide identity.

MPB Genome gene model ID	Accession number	Annotation	Females	Males	01	02	03	04	05	06	07	08	09	10	11	12	13	14
			Fold chge	Fold chge						JH		MT	JH	JH	Fed	Fed	JH +MT	cold
					Larvae	Pupae	Pupae	Adult ant.	Adult	Adult	Adult	Adult	Adult Mid	Adult Mid	Adult Mid	Adult Mid	Adult head	Larvae
augustus_masked-Seq_1102823-abinit-gene-33.19	YQE_09293	vitellogenin [Anthonomus grandis]	1472	–	–	–	–	–	–	2	–	–	12	4	3	–	2	–
genemark-Seq_1102823-abinit-gene-33.1	YQE_09290	vitellogenin [Anthonomus grandis]	1486	–	–	–	–	–	–	–	–	–	–	–	6	–	–	–

**Table 11:** Summary table for significantly (padj<0.01) increasing and decreasing transcripts implicated in pheromone flux in fed versus starved males and females including the number of reads in each EST library 01 to14 with greater than 99% nucleotide identity.

MPB Genome gene model ID	Accession number	Annotation	Females	Males	01	02	03	04	05	06	07	08	09	10	11	12	13	14
			Fold chge	Fold chge						JH		MT	JH	JH	Fed	Fed	JH +MT	cold
					Larvae	Pupae	Pupae	Adult ant.	Adult	Adult	Adult	Adult	Adult Mid	Adult Mid	Adult Mid	Adult Mid	Adult head	Larvae
maker-Seq_1101939-augustus-gene-14.30	YQE_02503	HMG-CoA reductase [Dendroctonus jeffreyi]	–	116.1	–	–	–	–	–	–	–	–	–	4	4	–	–	–
augustus_masked-Seq_1103039-abinit-gene-2.0	YQE_13014	HMG-CoA synthase [Dendroctonus jeffreyi]	–	4.4	–	–	–	–	–	–	4	7	8	2	–	3	–	7
augustus_masked-Seq_1102838-abinit-gene-1.7	YQE_09494	geranylgeranyl pyrophosphate synthase [Pediculus humanus corporis]	–	5.3	–	–	–	–	–	–	–	–	–	8	6	–	–	–

**Table 12:** Summary table for significantly (padj<0.01) increasing and decreasing plant cell wall degrading enzyme transcripts in fed versus starved males and females including the number of reads in each EST library 01 to14 with greater than 99% nucleotide identity.

MPB Genome gene model ID	Accession number	Annotation	Females	Males	01	02	03	04	05	06	07	08	09	10	11	12	13	14
			Fold chge	Fold chge						JH		MT	JH	JH	Fed	Fed	JH +MT	cold
					Larvae	Pupae	Pupae	Adult ant.	Adult	Adult	Adult	Adult	Adult Mid	Adult Mid	Adult Mid	Adult Mid	Adult head	Larvae
maker-Seq_1102288-augustus-gene-0.37	YQE_03146	endo-beta-1,4-glucanase [Dendroctonus ponderosae]	131.8	–	2	–	–	–	7	–	–	–	–	2	9	2	–	–
augustus_masked-Seq_1102656-abinit-gene-0.1	YQE_05116	endopolygalacturonase [Dendroctonus ponderosae]	88.9	–	–	–	–	–	–	–	–	–	–	2	10	–	–	–
maker-Seq_1102288-augustus-gene-0.38	YQE_03147	endo-beta-1,4-glucanase [Dendroctonus ponderosae]	42.0	–	–	–	–	–	6	–	–	–	4	4	4	–	–	2
augustus_masked-Seq_1098727-abinit-gene-0.0	YQE_00562	beta-1,3-glucanase [Tribolium castaneum]	–	32.4	2	–	–	–	4	2	4	2	24	4	6	10	–	8
maker-Seq_1102808-snap-gene-0.30	YQE_08964	endopolygalacturonase [Dendroctonus ponderosae]	27.8	–	–	–	–	–	–	–	–	–	2	–	–	–	–	–
snap_masked-Seq_1102734-abinit-gene-2.31	YQE_07359	pectin methylesterase [Dendroctonus ponderosae]	17.3	–	2	–	–	–	–	–	–	–	–	–	14	2	–	2
maker-Seq_1097454-snap-gene-0.20	YQE_00367	beta-1,3-glucanase [Tenebrio molitor]	–	10.3	–	–	2	–	2	–	–	–	–	–	19	–	–	–
maker-Seq_1102966-snap-gene-0.49	YQE_10920	beta-1,3-glucanase [Tenebrio molitor]	–	8.7	–	–	2	–	2	–	–	–	–	–	19	–	–	–
augustus_masked-Seq_1102571-abinit-gene-0.5	YQE_04715	beta-1,3-glucanase [Tribolium castaneum]	9.2	–	2	–	–	–	4	2	4	2	24	4	6	10	–	8
maker-Seq_1096691-augustus-gene-0.12	YQE_00223	pectin methylesterase [Dendroctonus ponderosae]	5.5	–	2	–	–	–	4	–	2	6	20	4	4	11	–	10
maker-Seq_1102656-augustus-gene-0.43	YQE_00223	endopolygalacturonase [Dendroctonus ponderosae]	4.9	–	–	–	–	–	–	–	–	–	–	–	9	1	–	2
maker-Seq_1101933-augustus-gene-0.55	YQE_02411	endopolygalacturonase [Dendroctonus ponderosae]	4.8	–	–	–	–	–	–	–	–	–	–	1	4	–	–	–
maker-Seq_1102656-augustus-gene-0.41	YQE_05115	endopolygalacturonase [Dendroctonus ponderosae]	4.3	–	–	–	–	–	–	–	–	–	–	1	4	–	–	–
augustus_masked-Seq_1103037-abinit-gene-8.8	YQE_12722	endo-beta-1,4-glucanase [Dendroctonus ponderosae]	3.3	–	–	2	–	–	–	7	12	4	47	14	14	33	–	9
maker-Seq_1102288-augustus-gene-0.39	YQE_03148	endo-beta-1,4-glucanase [Dendroctonus ponderosae]	–	2.8	2	4	7	–	10	11	19	16	71	6	13	37	–	2
maker-Seq_1098165-augustus-gene-0.14	YQE_00457	pectin methylesterase [Dendroctonus ponderosae]	2.8	–	2	–	–	–	4	–	2	6	20	4	4	11	–	10

## Discussion

We predicted that a number of gene families could be important in host chemical detoxification for the mountain pine beetle. Transcript levels for gene family members that differed significantly in either females or males fed with host tissue compared to starved insects included cytochromes P450, a glutathione S-tranferase, esterases, and one ABC transporter. Other transcripts that showed significant shifts in accumulation that have potential roles in detoxification of host defenses include alcohol dehydrogenases and some immune response genes as well as a group of unexpected gene transcripts that may play an, as yet, undiscovered role in host colonization by mountain pine beetle.

### Cytochromes P450

Insect cytochrome P450 enzymes have previously been implicated in the detoxification of exogenous compounds. Brattsten et al. [19] showed the induction of mixed function oxidase activity after southern armyworm (*Spodoptera eridania*) larvae exposure to conifer secondary metabolites. These enzymes are ubiquitous in nature; they are found in bacteria, plants, fungi, and animals. Cytochrome P450 enzymes have diverse functions, but in metazoan they are often involved in the oxygenation of xenobiotics thereby reducing toxicity or facilitate excretion by increasing hydrophilicity. In insects, cytochromes P450 perform a large array of detoxification reactions [20]. Bark beetles express functional cytochromes P450, and the expression and amount of some of these enzymes or their transcripts varies with feeding on host tissues [21]–[23] and with developmental stage [24]; or with treatment juvenile hormone levels [25] suggesting a potential role in metabolite detoxification. Cytochromes P450s are also involved in key physiological processes in bark beetles such as pheromone biosynthesis [26]–[28].

Among the sequences annotated as cytochromes P450 in the male mountain pine beetle genome [16], we identified six transcripts (Table 2) that significantly increased (four in females, one in males, and one in both) and five whose transcript levels significantly decreased following feeding in host phloem (three in females, one in males, and one in both) (Table 3). The cytochrome P450 transcripts that increased significantly with feeding in our experiments were also identified in EST libraries [29] generated from whole adults treated with terpenes and juvenile hormone, whereas those that decreased significantly were predominantly found in libraries originating from the head or antennal region (Table 3). Those that increased in the midgut, fatbody, and the whole adult insects and larvae are more likely to be involved in detoxification of ingested host plant secondary metabolites. One of the cytochrome P450 transcripts that decreased with feeding (CYP345E2) was found only in EST libraries derived from antennal-specific or head-specific tissue. This suggests that this cytochrome P450 is potentially involved in olfaction, a process that is more important during prior host colonization and mate selection prior to feeding.

**Figure 2:**
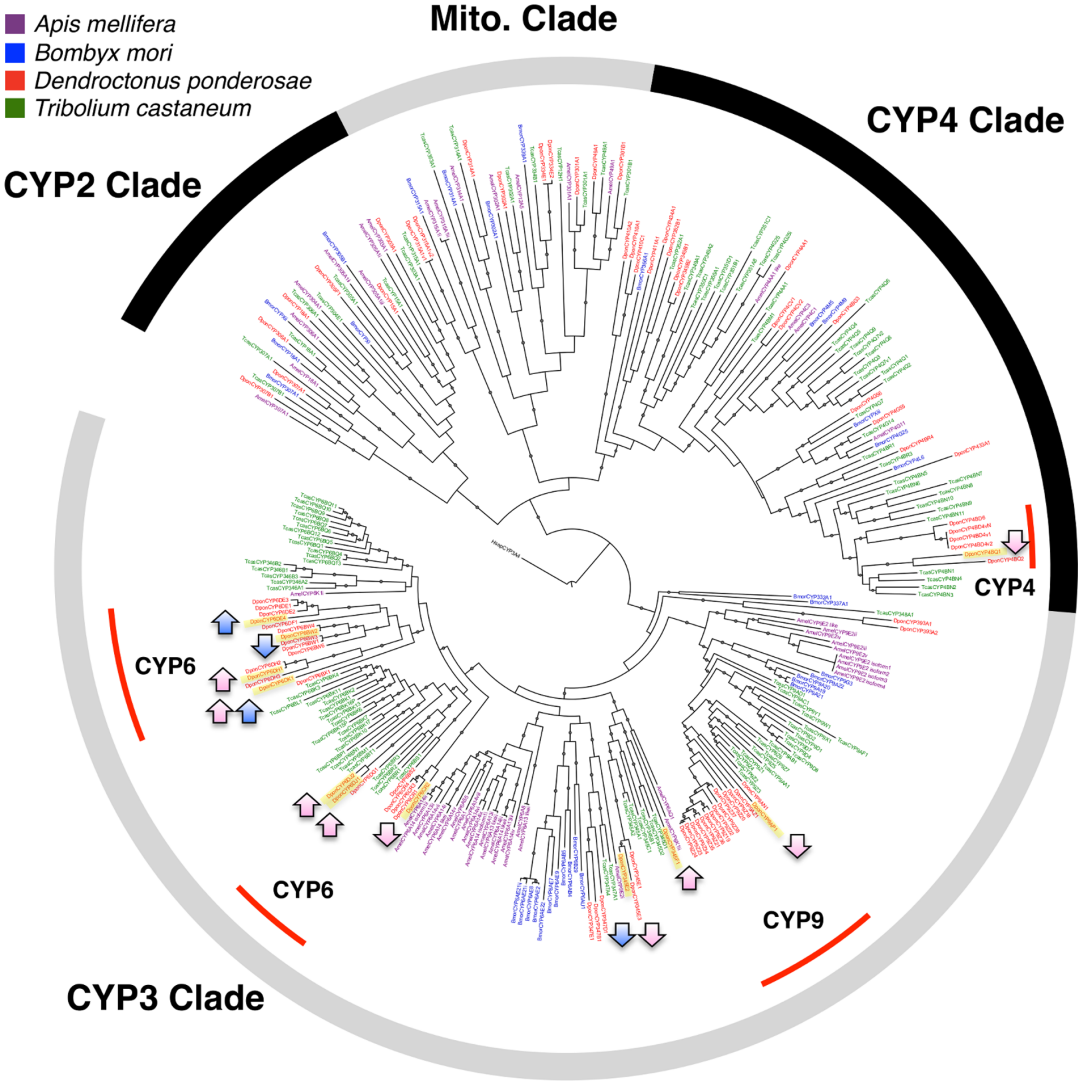
Identified Mountain Pine Beetle Cytochrome P450 Gene Transcripts. Phylogeny of all identified full-length cytochromes P450 identified in the mountain pine beetle genome as compared to cytochrome P450 sequences from the genomes of *Apis melifera*, *Bombyx mori*, and *Tribolium castaeum* (figure modified from 29). Significantly changing cytochrome P450 transcripts are highlighted, and the direction of change is designated by arrows; female transcripts are in pink, male transcripts are in blue.

Although function is almost impossible to predict from the sequence information of a cytochrome P450 gene alone [30], comparison of phylogenies in addition to information of expression in different tissues or under different treatment conditions, may suggest reasonable hypotheses for functional characterization efforts. The annotated cytochrome P450 transcripts identified here align to full-length cDNAs of mountain pine beetle cytochromes P450 identified in [29] and [16]. The cytochromes P450 from our study fall into several CYP families including CYP4, CYP6, and CYP9 (Figure 2). Of the cytochrome P450 transcripts that increased significantly with feeding, four showed a female-specific change (CYP6DH1, CYP345F1, CYP6DJ1, and CYP6DJ2), one showed a male-specific change (CYP6DE4), and one increased significantly in both sexes (CYP6DK1). Of the cytochrome P450 transcripts that decreased significantly with feeding, three of the five showed female-specific changes (CYP6CR2, CYP4BQ1, CYP9AP1), one showed a male-specific shift (CYP6BW2), and one decreased significantly in both sexes (CYP345E2) (Table 3).

Host tree defense compounds are primary substrate candidates for the functional characterization of up-regulated cytochromes P450. These include terpenoid compounds and phenolics. Sex specific up-regulation of cytochromes P450 suggests a detoxification mechanism that is linked to other sex-specific physiological processes, for example in the production of hormones or pheromones for excretion. Recently, changes in transcripts annotated as cytochrome P450s in the closely related *Dendroctonus spp.* midguts, fatbodies, and antennae were shown to increase in response to common conifer host monoterpenes [31]–[32]. Our data showed a large significant female-specific increase in CYP6DH1. As female beetles are the pioneers (females initiate attacks, excavate the initial galleries, then are joined by the male beetles), our data suggest a possible increased importance of female detoxification of components of oleoresin in the first 24 hours after colonization. As the males show the largest increase in CYP6DE4 shortly after colonization, functional characterization of this enzyme would shed further light on the host colonization roles and physiological activities of males in the first 24 hours after attack.

### Glutathione S-transferases (GST) and Glucosyl/Glucuronosyl Transferases

GSTs and glucosyl or glucuronosyl transferases are a large group of enzymes that transfer a glutathione (or glucosyl, or glucuronsyl) moiety to a variety of substrates. GSTs, in particular, have been implicated in the detoxification of insecticides containing organophosphates [33], organochlorines [34]–[35], and pyrethroids [36]–[37]. GSTs conjugate xenobiotic compounds with a glutathione moiety (GSH) and often work in tandem with cytochromes P450 or other enzymes [38] that aid in detoxification, sequestration or excretion of toxic compounds. GSTs have been previously cloned from spruce budworm, *Choristoneura fumiferana*, an insect that also feeds on conifer tissue [39], and the mountain pine beetle GSTs have been annotated in EST libraries [29] and in the mountain pine beetle genome [16]. We identified three significantly upregulated transcripts annotated as glucosyl/glucuronosyl transferases in the feeding females, and two are significantly downregulated (**Table 4**). In males, five transcripts annotated as glucosyl/glucuronosyl transferases increased significantly with feeding, and none were found to significantly decrease (**Table 4**). Three of the up-regulated transferase transcripts increased in both males and females with very similar fold changes. One of these transcripts annotated as a GST (snap_masked-Seq_1102275-abinit-gene-0.28, DpGSTs2) matched EST database sequences extracted from the midgut and fatbody of fed adult beetles. This putative GST is a good candidate for further study on mountain pine beetle detoxification mechanisms.

### Esterases

Esterases are a large class of enzymes that are also implicated in insect resistance to insecticides and other xenobiotics. The up-regulation and mutation of esterase genes has been associated with insecticide resistance in insect orders such as Hymenoptera (wasps), Lepidoptera (moths), and Diptera (flies) (reviewed in [40]). We identified two esterase gene models (genemark-Seq_1102891-abinit-gene-0.13 and maker-Seq_1102774-snap-gene-21.56) that were significantly up-regulated in fed females and that may have roles in host chemical detoxification. We also observed two esterase gene transcripts (maker-Seq_1102308-snap-gene-6.52 and maker-Seq_1103039-snap-gene-0.57) that decreased significantly in female beetles allowed to feed on host tissues. Transcripts that decrease shortly after host colonization could be associated with physiological processes such as odorant degradation that are more important prior to entry into the host tree.

### ABC Transporters

ABC transporters – a large class of proteins best known for multi-drug resistance in humans and *Drosophila melanogaster* [41] and *Anopheles gambiae* [42] – have been shown to aid in the sequestration and elimination of toxic xenobiotic compounds in Lepidopteran insects [43], and may perform a similar function in the mountain pine beetle. Although we identified 12 transcripts that may be important for signaling and transport mechanisms in females allowed to feed on host tissue (Figure 2), transcript levels of only one ABC transporter (maker-Seq_1102955-augustus-gene-1.33) significantly increased in females beetles feeding on host tissue (Table 6). Evidence for this transcript could only be found in the EST libraries (library DPO11) originating from combined midgut/fatbody tissue of beetles feeding on host tissue [29]. We hypothesize that this gene is involved in digestion and detoxification of the ingested, resin-saturated phloem that is present in the tree after the initial attack [12].

### Alcohol Dehydrogenases

The transcripts of three putative alcohol dehydrogenases increased significantly in females feeding on host tissue and transcripts for two alcohol dehydrogenases increased significantly in males. In total, four transcripts increased following feeding on host tissue in the two sexes with one (maker-Seq_1093767-snap-gene-0.4) that increased in both males and females. None of the transcripts that decreased significantly in males or females were annotated as alcohol dehydrogenases. Alcohol dehydrogenases may play a role in metabolizing terpenoid alcohols that are a component of lodgepole pine oleoresin [44], and bark beetles are able to sense and respond to terpene alcohols [45]. For example, 3-caren-10-ol altered the sex specificity of attraction to pheromone bait, and the terpene composition of the host tree determined some of the pheromone production during host colonization [46]. Terpene alcohols also impact other coniferophagous beetles, for example the volatile monoterpene, linalool, that is produced *de novo* by Sitka spruce in response to attack by white pine weevil [47]. Conifers contain a large number of metabolites with alcohol functional groups including ethanol and phenolic compounds that are produced after mountain pine beetle attack [48]. Finally, alcohol dehydrogenases have been functionally characterized in essential bark beetle metabolic processes. For example, ipsdienol dehydrogenase (IDOLDH) acts on hydroxylated myrcene to produce ipsdienol, an important aggregation pheromone in *Ips pini* to produce ipsdienone [49]. As transcripts for alcohol dehydrogenases showed general increases, and none decreased in our study, this group of enzymes warrants further study for its role in the detoxification of host specialized metabolites in MPB.

### Oxidative Stress, Damage Control, and Immune Response

A number of transcripts were identified by their annotations to have a likely role in stress physiology, damage control, and an immune response after host colonization.

#### Oxidative stress and damage control

Superoxide dismutases function as antioxidants breaking down superoxides (reactive oxygen species, ROS) into hydrogen peroxide and water. They are therefore important regulators of ROS and are implicated in the reduction of oxidative damage [50]. Transcript levels for one superoxide dismustase decreased significantly in males and females feeding on host tissue (maker-Seq_1102995-snap-gene-0.45) (Table 7). There were also minor, yet significant, increases in proteasome subunit beta and thioredoxin-like transcripts in males, as well as in DNA damage inducible proteins in females, suggesting some level of oxidative stress or signaling by reactive oxygen species after exposure to host tissues. In our data, adult beetles show a more pronounced shift toward reproduction and detoxification by cytochromes P450 and GST enzymes; the more subtle changes in other transcripts may represent a shift from survival to senescence for adult beetles as they near the usual completion of their life cycle following successful reproduction.

#### Luciferin-regenerating enzyme

Although this enzyme occurs in many insects, it is most commonly known to be involved in bioluminescence in two families of the Elateroidea (Coleoptera) – specifically the Lampyridae (fireflies) and the Phengodidae (glowworm beetles) [51]. Bioluminescence in these organisms occurs when luciferase oxidizes the luciferin substrate to produce oxyluciferin. The luciferin-regenerating enzyme then catalyzes a two-step reaction to regenerate luciferin and emit light [52]. Both a luciferase-like gene sequence and a luciferin-regenerating enzyme-like sequence have been annotated in the mountain pine beetle genome [16]. The luciferin-regenerating enzyme was significantly upregulated in females in our experiments (Table 7). There are currently no known examples of bark beetles communicating using bioluminescence, but it is possible that these enzymes may be involved in communication in low light conditions present under the bark. Neo- or sub-functionalization of a common beetle transcript towards detoxification or alteration of new substrates could also occur for an enzyme specializing in substrate oxidation. Because luciferase activity is ubiquitously associated with reactive oxygen species as a source of molecular oxygen transferred to luciferin, Day et al. [51] note that this type of bioluminescence in insects may have evolved from an early mechanism to detoxify ROS [53]–[55] although they take care to point out that bioluminescence is not necessarily the only evolutionary prerequisite for ROS detoxification. The luciferin-regenerating enzyme is a candidate for several potential physiological roles for female beetles during early host colonization.

#### Anti-viral/anti-microbial immune response

Anti-viral transcripts may represent an induced response to new host tissue, a virus present in the new host, or a response to exposure to a virus that was present in their previous life history, for example, by exposure to other con- or heterospecific associates in the brood tree. In our data, the largest change in this category occurred in females for a transcript annotated as a salivary c-type lectin (Table 9). C-type lectins are a large and widespread group of animal proteins that play a key role in the innate immune response by facilitating the recognition of common molecular patterns in pathogens; they have also been described in insects [56]. A second group of transcripts annotated as scavenger receptors are also a broad group of proteins in the animal innate immune response that specialize in removal of bacteria and apoptitic cells. In *Drosophila* cell lines, scavenger receptors are associated with macrophage endocytosis of dead and foreign cells [57]. Other examples with smaller fold changes in females following feeding include transcripts annotated as a lysostaphin – an enzyme that cleaves pentaglycin bridges in *Staphylococcus spp.* [58] – as well as an EBNA (Epstein-barr nuclear antigen) binding protein and an IFN (interferon-mediated) anti-viral response transcript. The latter two are expressed in response to viral infection in mammals, although we could not find evidence in the literature of examples in insects.

### Reproduction, Pheromone Flux, and Digestion

Other metabolic processes evident from shifts in transcript levels following feeding include the production of vitellogenin precursors in preparation for a metabolic switch to reproduction in females, the production of the peritrophic matrix involved in digestion, the down-regulation of gene transcripts annotated as key enzymes in the citric acid cycle, and a reduction in the enzymes required for fatty acid synthesis.

#### Reproduction

Vitellogenin, an egg provisioning precursor lipoglycoprotein, emerged as having the most highly differentially expressed transcript between starved females and females exposed to host tissues. The vitellogenin transcripts showed over a 1400-fold increase in expression over the starved control females (Table 10). This demonstrates a shift in female physiology reallocating resources to the production of eggs. Vitellogenin has also been shown to act as an antioxidant in honeybees [59]. As transcripts annotated as vitellogenin were also found in male mountain pine beetles (although not changing significantly in this experiment), production of this transcript in response to some oxidative stress resulting from a new and defended host tree could play a minor role in the highly differential expression in female beetles. Such a highly significant change in vitellogen transcript expression suggests that females quickly allocate resources to reproduction only after entering a susceptible host, this supports early data on the physical changes, including muscle degradation and egg production that occurs in beetles after host colonization [60]. Finally, these data also suggests that substantial flight exercise is not required for a rapid switch to reproductive physiology when a susceptible host is encountered, as we did not allow beetle flight between emergence and experimental treatment.

#### Pheromone flux

*exo*-Brevicomin, a male produced pheromone, is hypothesized to be formed from the fatty acid synthesis pathway [61]–[62], and thus the reduction in gene transcripts involved in fatty acid synthesis may represent a shift from pheromone production to facilitate mass attack to feeding and reproduction, especially in male beetles. As *exo*-brevicomin levels are reduced when male beetles enter the tree and a shift to the *de novo* production of frontalin [63] occurs, we would expect an increase in the transcripts from mevalonate pathway. Our data showed male-specific increases in 3-hydroxy-3-methylglutaryl-CoA synthase and 3-hydroxy-3-methylglutaryl-CoA reductase transcripts, key enzymes in the mevalonic pathway, as well as an increase in a putative geranylgeranyl pyrophosphate synthase transcript (Table 11). As mountain pine beetle pheromones are formed from isoprenoid precusors [64], an increase in these enzymes that are involved in the production of the isoprenoid skeleton supports the production of frontalin by host colonizing males. Females do not show a change in transcript accumulation for 3-hydroxy-3-methylglutaryl-CoA synthase and 3-hydroxy-3-methylglutaryl-CoA reductase, and this is supported by similar expression pattern data from the German cockroach [65]. Aw et al. [21] did not observe a decrease in transcripts associated with fatty acid metabolism between male beetles before and after entry into a host tree, nor did they observe a change in mevalonate pathway genes in their study. However, their studies were conducted using microarray technology that the authors suggest may not detect changes in expression where few EST are sequenced [21] [7]. They did, however, also detect a change in a transcript annotated as a geranylgeranyl pyrophosphate synthase that they suggest may be involved in frontalin synthesis as well [21].

#### Plant cell wall degrading enzymes (PCWDE)

Plant cell wall degrading enzymes are comprised of a large group of enzymes that aid in the metabolism of plant cell walls and, they are predicted to occur in high diversity in beetles [66], and are abundantly annotated in the mountain pine beetle genome [16]. In general, there is a large and varied increase in the expression of PCWDE’s in fed versus starved beetles (Table 12) highlighting the importance of cell wall digestion at this early stage of colonization in both sexes.

## Conclusions

Expression analysis by large-scale sequencing of the transcriptome allows for low-bias identification of potentially important genes and gene families involved in various physiological shifts during mountain pine beetle host colonization and early reproduction. Not only will this study build on a wealth of currently available genomics resources becoming available for the mountain pine beetle [16] [29] [24] [21], this analysis of the transcriptome during early host colonization begins the task of associating genes and proteins with the larger implications of insect outbreaks on ecosystems. This molecular-level study on insect metabolism of host metabolites provides new information on the ability of mountain pine beetle to cope with toxic host defenses and may ultimately help to predict the extent and rate of beetle population expansion into new hosts – for instance jack pine, *Pinus banksiana* (1) and whitebark pine, *Pinus albicaulis* [67] – in Canada’s boreal forests.

## Supporting Information

Table S1 **Parameters and setting used to map RNA-seq data onto the gene models of the male mountain pine beetle genome.**
(DOCX)

## References

[1] Cullingham CI, Cooke JE, Dang S, Davis CS, Cooke BJ, et al (2011) Mountain pine beetle host-range expansion threatens the boreal forest. Mol Ecol. 20(10): 2157–71 10.1111/j.1365-294X.2011.05086.x PMC3116149 21457381

[2] Nealis V, Peter B (2009) Risk assessment of the threat of mountain pine beetle to Canada’s boreal and eastern pine forests. Canadian Forest Service Information Report BC-X-417. Victoria, British Columbia.

[3] Raffa KF, Aukema BH, Bentz BJ, Carroll AL, Hicke JA, et al (2008) Cross-scale drivers of natural disturbances prone to anthropogenic amplification: The dynamics of bark beetle eruptions. BioSci. 58: 501–517.

[4] British Columbia Ministry of Lands, Forests, and Natural Resource Operations (2012) A History of the Battle Against the Mountain Pine Beetle website. Available: http://www.for.gov.bc.ca/hfp/mountain_pine_beetle/Pine%20Beetle%20Response%20Brief%20History%20May%2023%202012.pdf Accessed 2013 Jan 07.

[5] Schneider RR, Latham MC, Stelfox B, Farr D, Boutin S (2010) Effects of a severe mountain pine beetle epidemic in western Alberta, Canada under two forest management scenarios. Intl. J. For. Res. Article ID 417595 doi:10.1155/2010/417595.

[6] Volney WJA, Fleming RA (2000) Climate change and impacts of boreal forest insects. Agri. Ecosys. Environ. 82(1–3): 283–294.

[7] Keeling CI, Bohlmann J (2006) Tansley Review: Genes, enzymes, and chemicals of terpenoid diversity in the constitutive and induced defence of conifers against insects and pathogens. New Phytol. 170(4): 657–75. 10.1111/j.1469-8137.2006.01716.x 16684230

[8] Raffa KF, Berryman AA (1982) Physiological differences between lodgepole pines resistant and susceptible to the mountain pine beetle and associated microorganisms. Environ. Entomol. 11(2): 486–492.

[9] Diguistini S, Wang Y, Liao N, Taylor G, Tanguay P, et al (2011) Genome and transcriptome analyses of the mountain pine beetle-fungal symbiont *Grosmannia clavigera*, a lodgepole pine pathogen. Proc. Natl. Acad. Sci. U S A. 108(6): 2504–9 10.1073/pnas.1011289108 PMC3038703 21262841

[10] Wang Y, Lim L, DiGuistini S, Robertson G, Bohlmann J, et al (2013) A specialized ABC efflux transporter GcABC-G1 confers monoterpene resistance to *Grosmannia clavigera*, a bark beetle-associated fungal pathogen of pine trees. New Phytol. 197(3): 886–98 10.1111/nph.12063 23252416

[11] Sangwon L, Kim JJ, Breuil C (2006) Pathogenicity of *Leptographium longiclavatum* associated with *Dendroctonus ponderosae* to *Pinus contorta*. Can. J. For. Res. 36: 2864–2872.

[12] Clark EL, Huber DPW, Carroll AL (2012) The legacy of attack: implications of high phloem resin monoterpene levels in lodgepole pines following mass attack by mountain pine beetle, *Dendroctonus ponderosae* Hopkins. Environ. Entomol. 41(2): 392–8 10.1603/EN11295 22507014

[13] Lyon RL (1958) A useful secondary sex character in *Dendroctonus* bark beetles. Can. Entomol. 90: 582–584.

[14] Winnebeck EC, Millar CD, Warman GR (2010) Why does insect RNA look degraded? J. Insect. Sci. 10: 159. 10.1673/031.010.14119 PMC3016993 21067419

[15] Fraser JD (2011) Cold tolerance and seasonal gene expression in *Dendroctonus ponderosae* [M.Sc. thesis] University of Northern British Columbia Faculty of Natural Resource and Environmental Studies, The University of Northern British Columbia Library website. Available: https://encore.unbc.ca/iii/encore/record/C__Rb1741108. Accessed 2013 Jan 07.

[16] Keeling CI, Yuen MMS, Liao NY, Docking TR, Chan SK, et al (2013) Draft genome of the mountain pine beetle, *Dendroctonus ponderosae* Hopkins, a major forest pest. Genome Biol. 14(3): R27. 10.1186/gb-2013-14-3-r27 PMC4053930 23537049

[17] Anders S, Huber W (2010) Differential expression analysis for sequence count data. Genome Biol. 11(10): R106 10.1186/gb-2010-11-10-r106 PMC3218662 20979621

[18] Benjamini Y, Hochberg Y (1995) Controlling the False Discovery Rate: a Practical and Powerful Approach to Multiple Testing. Journal of the Royal Statistical Society. Series B (Methodological). 57(1): 289–300.

[19] Brattsten LB, Wilkinson CF, Eisner T (1977) Herbivore-plant interactions: mixed-function oxidases and secondary plant substances. Science 196(4296): 1349–52. 17831753 10.1126/science.196.4296.1349

[20] Li X, Baudry J, Berenbaum MR, Schuler MA (2004) Structural and functional divergence of proteins: From specialist to generalist cytochrome P450. Proc. Natl. Acad. Sci. U S A. 101(9): 2939–44. 10.1073/pnas.0308691101 PMC365724 14981232

[21] Aw T, Schlauch K, Keeling CI, Young S, Bearfield JC, et al (2010) Functional genomics of mountain pine beetle (Dendroctonus ponderosae) midguts and fat bodies. BMC Genomics 11: 215 10.1186/1471-2164-11-215 20353591 PMC2858752

[22] Huber DPW, Erickson ML, Leutenegger CW, Bohlmann J, Seybold SJ (2007) Isolation and extreme sex-specific expression of cytochrome P450 genes in the bark beetle, *Ips paraconfusus*, following feeding on the phloem of host ponderosa pine, *Pinus ponderosa*. Insect Mol. Biol. 16(3): 335–49. 10.1111/j.1365-2583.2007.00731.x 17433069

[23] Tittiger C, Keeling CI, Blomquist GJ (2005) Some insights into the remarkable metabolism of the bark beetle midgut, Recent Adv. Phytochem. 39: 57–78.

[24] Bonnett T, Robert JA, Pitt C, Fraser JD, Keeling CI, et al (2012) Global and comparative proteomic profiling of overwintering and developing mountain pine beetle, *Dendroctonus ponderosae* (Coleoptera: Curculionidae), larvae. Insect Biochem. Mol. Biol. 42(12): 890–901 10.1016/j.ibmb.2012.08.003 22982448

[25] Keeling CI, Bearfield JC, Young S, Blomquist GJ, Tittiger C (2006) Effects of juvenile hormone on gene expression in the pheromone-producing midgut of the pine engraver beetle, *Ips pini.* Insect Mol. Biol 15 2: 207–16. 10.1111/j.1365-2583.2006.00629.x 16640731

[26] Song M, Kim AC, Gorzalski AJ, Maclean M, Young S, et al (2013) Functional characterization of myrcene hydroxylases from two geographically distinct *Ips pini* populations. Insect Biochem. Mol. Biol. 43 4: 336–43 10.1016/j.ibmb.2013.01.003 23376633

[27] Blomquist GJ, Figueroa-Teran R, Aw M, Song M, Gorzalski A, et al (2010) Pheromone production in bark beetles. Insect Biochem. Mol. Biol. 40(10): 699–712 10.1016/j.ibmb.2010.07.013 20727970

[28] Sandstrom P, Welch WH, Blomquist GJ, Tittiger C (2006) Functional expression of a bark beetle cytochrome P450 that hydroxylates myrcene to ipsdienol. Insect Biochem. Mol. Biol. 36(11): 835–45. 10.1016/j.ibmb.2006.08.004 17046597

[29] Keeling CI, Henderson H, Li M, Yuen M, Clark EL, et al (2012) Transcriptome and full-length cDNA resources for the mountain pine beetle, *Dendroctonus ponderosae* Hopkins, a major insect pest of pine forests. Insect Biochem. Mol. Biol. 42(8): 525–36 10.1016/j.ibmb.2012.03.010 22516182

[30] Feyereisen R (1999) Insect P450 enzymes. Annu. Rev. Entomol. 44: 507–33. 10.1146/annurev.ento.44.1.507 9990722

[31] López MF, Cano-Ramirez C, Cesar-Ayala AK, Ruiz EA, Zúñiga G (2013) Diversity and expression of P450 genes from *Dendroctonus valens* LeConte (Curculionidae: Scolytinae) in response to different kairomones. Insect Biochem. Mol. Biol. 43(5): 417–32 10.1016/j.ibmb.2013.02.004 23454142

[32] Cano-Ramirez C, Lopez MF, Cesar-Ayala AK, Pineda-Martinez V, Sullivan BT, et al (2012) Isolation and expression of cytochrome P450 genes in the antennae and gut of pine beetle *Dendroctonus rhizophagus* (Curculionidae: Scolytinae) following exposure to host monoterpenes. Gene 520(1): 47–63 10.1016/j.gene.2012.11.059 23262344

[33] Huang HS, Hu NT, Yao YE, Wu CY, Chiang SW, et al (1998) Molecular cloning and heterologous expression of a glutathione S-transferase involved in insecticide resistance from the diamondback moth, *Plutella xylostella*. Insect Biochem. Mol. Biol. 28(9): 651–8. 10.1016/s0965-1748(98)00049-6 9755475

[34] Ranson H, Rossiter L, Ortelli F, Jensen B, Wang X, et al (2001) Identification of a novel class of insect glutathione S-transferases involved in resistance to DDT in the malaria vector *Anopheles gambiae*. Biochem. J. 359(Pt 2): 295–304. 10.1042/0264-6021:3590295 PMC1222147 11583575

[35] Syvanen M, Zhou Z, Wharton J, Goldsbury C, Clark A (1996) Heterogeneity of the glutathione transferase genes encoding enzymes responsible for insecticide degradation in the housefly. J. Mol. Evol. 43: 236–240. 10.1007/BF02338831 8703089

[36] Vontas JG, Small GJ, Nikou D, Ranson H, Hemingway J (2002) Purification, molecular cloning and heterologous expression of a GST involved in insecticide resistance from the rice brown planthopper, *N. lugens*, Biochem. J. 362: 329–337. 10.1042/0264-6021:3620329 PMC1222392 11853540

[37] Vontas JG, Small GJ, Hemingway J (2001) Glutathione S-transferases as antioxidant defence agents confer pyrethroid resistance in *N. lugens*. Biochem. J. 357: 65–72. 10.1042/0264-6021:3570065 PMC1221929 11415437

[38] Sheehan D, Meade G, Foley VM, Dowd CA (2001) Structure, function and evolution of glutathione transferases: implications for classification of non-mammalian members of an ancient enzyme superfamily. Biochem. J. 360(Pt 1): 1–16. 10.1042/0264-6021:3600001 PMC1222196 11695986

[39] Feng Q, Davey KG, SD Pang A, Ladd TR, Retnakaran A, et al (2001) Developmental expression and stress induction of glutathione S-transferase in the spruce budworm. *Choristoneura fumiferana*. J. Insect Physiol. 47(1): 1–10. 10.1016/s0022-1910(00)00093-7 11033162

[40] Li X, Schuler MA, Berenbaum MR (2007) Molecular mechanisms of metabolic resistance to synthetic and natural xenobiotics. Annu. Rev. Entomol. 52: 231–53. 10.1146/annurev.ento.51.110104.151104 16925478

[41] Dean M, Rzhetsky A, Allikmets R (2001) The human ATP-binding cassette (ABC) transporter superfamily. Genome Res. 11(7): 1156–66. 10.1101/gr.184901 11435397

[42] Roth CW, Holm I, Graille M, Dehoux P, Rzhetsky A, et al (2003) Identification of the *Anopheles gambiae* ATP-binding cassette transporter superfamily genes. Mol. Cells. 15(2): 150–158. 12803476

[43] Labbé R, Caveney S, Donly C (2010) Genetic analysis of the xenobiotic resistance-associated ABC gene subfamilies of the Lepidoptera. Insect Mol. Biol. 20(2): 243–56 10.1111/j.1365-2583.2010.01064.x 21199020

[44] von Rudloff E, Lapp MS (1987) Chemosystematic studies in the genus *Pinus.* VI. General survey of the leaf oil terpene composition of lodgpole pine. Can. J. For. Res. 17: 1013–1025.

[45] Pureswaran DS, Gries R, Borden JH (2004) Antennal responses of four species of tree-killing bark beetles (Coleoptera: Scolytidae) to volatiles collected from beetles, and their host and nonhost conifers. Chemoecology. 14: 59–66.

[46] Libby LM, Ryker LC, Yandell KL (1985) Laboratory and field studies of volatiles released by *Dendroctonus ponderosae* Hopkins (Coleoptera, Scolytidae). Seitschrift fur Angewandte Entomologie. 100: 381–392.

[47] Miller B, Madilao LL, Ralph S, Bohlmann J (2005) Insect-induced conifer defense. White pine weevil and methyl jasmonate induce traumatic resinosis, *de novo* formed volatile emissions, and accumulation of terpneoid synthase and putative octadecanoid pathway transcripts in Sitka spruce. Plant Physiol. 137(1): 369–82. 10.1104/pp.104.050187 PMC548866 15618433

[48] Shrimpton DM (1973) Extractives associated with wound response of lodgepole pine attached by the mountain pine beetle and associated microorganisms. Can. J. Bot. 51: 527–534.

[49] Figueroa-Teran R, Welch WH, Blomquist GJ, Tittiger C (2012) Ipsdienol dehydrogenase (IDOLDH): a novel oxidoreductase important for *Ips pini* pheromone production. Insect Biochem. Mol. Biol. 42(2): 81–90 10.1016/j.ibmb.2011.10.009 22101251

[50] Landis GN, Tower J (2005) Superoxide dismutase evolution and life span regulation. Mech. Ageing Dev. 126(3): 365–79. 10.1016/j.mad.2004.08.012 15664623

[51] Day JC, Tisi LC, Bailey ML (2004) Evolution of bioluminescence: the origin of beetle luciferin. Luminescence. 19(1): 8–20. 10.1002/bio.749 14981641

[52] Gomi K, Kajiyama N (2001) Oxyluciferin, a luminescence product of firefly luciferase, is enzymatically regenerated into luciferin. J. Biol. Chem. 276(39): 36508–13. 10.1074/jbc.M105528200 11457857

[53] Barros MP, Bechara EJ (1998) Bioluminescence as a possible auxillary oxygen detoxifying mechanism in elaterid larvae. Free Radic. Biol. Med. 24(5): 767–77. 10.1016/s0891-5849(97)00335-3 9586807

[54] Barros MP, Becharra EJ (2000) Luciferase and urate may act as antioxidant defenses in larval *Pyrearinus termitilluminans* (Elateridae: Coleoptera) during natural development and upon 20-hydroxyecdysone treatment. Photochem. Photobiol. 71(5): 648–54. 10.1562/0031-8655(2000)071<0648:laumaa>2.0.co;2 10818797

[55] Barros MP, Becharra EJ (2001) Daily variations of anti oxidant enzyme and luciferase activities in the luminescent click-beetle *Pyrearinus termitilluminans*: cooperation against oxygen toxicity. Insect. Biochem. Mol. Biol. 31(4–5): 393–400. 10.1016/s0965-1748(00)00132-6 11222948

[56] Watanabe A, Miyazawa S, Kitami M, Tabunoki H, Ueda K, et al (2006) Characterization of a novel C-type lectin, *Bombyx mori* multibinding protein, from the *B. mori* hemolymph: Mechanisms of wide-range microorganism recognition and role in immunity. J Immunol. 177(7): 4594–604. 10.4049/jimmunol.177.7.4594 16982897

[57] Abrams JM, Lux A, Steller H, Krieger M (1992) Macrophages in Drosophila embryos and L2 cells exhibit scavenger receptor-mediated endocytosis. Proc. Natl. Acad. Sci. U S A. 89(21): 10375–9. 10.1073/pnas.89.21.10375 PMC50341 1438223

[58] Schindler CA, Schuhardt VT (1964) Lysostaphin: A new bacteriolytic agent for the *Staphylococcus*. Proc. Natl. Acad. Sci. U S A. 51: 414–21. 10.1073/pnas.51.3.414 PMC300087 14171453

[59] Seehuus SC, Norberg K, Gimsa U, Krekling T, Amdam GV (2006) Reproductive protein protects functionally sterile honey bee workers from oxidative stress. Proc. Natl. Acad. Sci. U S A. 103(4): 962–7. 10.1073/pnas.0502681103 PMC1347965 16418279

[60] Reid RW (1958) Internal changes in female mountain pine beetle, *Dendroctonus monticolae* Hopk., associated with egg laying and flight. Can. Entomol. 90(8): 464–468.

[61] Francke W, Schroder F, Philipp P, Meyer H, Sinnwell V, et al (1996) Identification and synthesis of new bicyclic acetals from the mountain pine beetle, *Dendroctonus ponderosae* Hopkins (Col.: Scol.). Bioorg. Med. Chem. 4(3): 363–74. 10.1016/0968-0896(96)00013-2 8733614

[62] Vanderwel D, Gries G, Singh SM, Borden JH, Oehlschlager AC (1992) (E)- and (Z)-6-nonen-2-one: biosynthetic precursors of endo - and exo-brevicomin in two bark beetles (Coleoptera: Scolytidae). J. Chem. Ecol. 18: 1389–1404. 10.1007/BF00994364 24254214

[63] Barkawi LS, Francke W, Blomquist GJ, Seybold SJ (2003) Frontalin: *De novo* biosynthesis of an aggregation pheromone component by *Dendroctonus* spp. bark beetles (Coleoptera: Scolytidae). Insect Biochem. Mol. Biol. 33(8): 773–88. 10.1016/s0965-1748(03)00069-9 12878224

[64] Gilg AB, Bearfield JC, Tittiger C, Welch WH, Blomquist GJ (2005) Isolation and functional expression of the first animal geranyl diphosphate synthase and its role in bark beetle pheromone biosynthesis. Proc. Natl. Acad. Sci. U S A. 102(28): 9760–5. 10.1073/pnas.0503277102 PMC1174994 15983375

[65] Buesa C, Martinez-Gonzalez J, Casals N, Haro D, Piulachs MD, et al (1994) *Blattella germanica* has two HMG-CoA synthase genes. Both are regulated in the ovary during the gonadotrophic cycle. J. Biol. Chem. 269(16): 11707–13. 7909314

[66] Pauchet Y, Wilkinson P, Chauhan R, ffrench-Constant RH (2010) Diversity of beetle genes encoding novel plant cell wall degrading enzymes. PLoS One 5(12): e15635 10.1371/journal.pone.0015635 21179425 PMC3003705

[67] Raffa KF, Powell EN, Townsend PA (2012) Temperature-driven range expansion of an irruptive insect heightened by weakly coevolved plant defenses. Proc. Natl. Acad. Sci. U S A. 110(6): 2193–8 10.1073/pnas.1216666110 PMC3568305 23277541

