# Supplementary material for: Disentangling Detoxification: Gene Expression Analysis of Feeding Mountain Pine Beetle Illuminates Molecular-Level Host Chemical Defense Detoxification Mechanisms
Source: PLoS One. 2013 Nov 1;8(11):e77777. doi: 10.1371/journal.pone.0077777 (PMC3815198; doi:10.1371/journal.pone.0077777)
Supplement: Table S1 — Parameters and setting used to map RNA-seq data onto the gene models of the male mountain pine beetle genome. (DOCX) [file pone.0077777.s001.docx]

Supplementary Information

| **Parameter** | **Setting** |
| --- | --- |
| Create fusion gene table | No |
| Create report | Yes |
| Create list of unmapped reads | No |
| Additional downstream bases | 2000 |
| Exon discovery | No |
| Minimum read count fusion gene table | 5 |
| Maximum paired distance | 400 |
| Minimum paired distance | 100 |
| Minimum length of putative exons | 50 |
| Minimum number of reads | 10 |
| Maximum number of mismatches (short reads) | 2 |
| Organism type | Eukaryote |
| Use annotations for gene and transcript identification | Yes |
| Expression level possible values: Genes, Transcripts | Genes |
| Unspecific match limit | 10 |
| Additional upstream bases | 2000 |
| Count paired reads as two | No |
| Use colorspace encoding | No |
| Minimum exon coverage fraction | 0.2 |
| Minimum length fraction (long reads) | 0.8 |
| Minimum similarity fraction (long reads) | 0.8 |
| Expression value | Read Per Kilobase of exon Model value |
